# The role of bidirectional associations between depression, anxiety, and emotional exhaustion on turnover intention among nurses: a multicenter cross-sectional study in China

**DOI:** 10.1186/s12912-023-01516-1

**Published:** 2023-10-03

**Authors:** Ning Qin, Ziqiang Yao, Meiying Guo

**Affiliations:** 1 Nursing Department, The Third Xiangya Hospital, Central South University, Changsha, Hunan China; 2 Xiang Ya Nursing School, Central South University, Changsha, Hunan China

**Keywords:** Turnover intention, Depression, Anxiety, Emotional exhaustion, Nurses, Bidirectional associations, Medication effect

## Abstract

**Background:**

A high turnover rate in nursing has become a global concern. Mental health issues may increase the turnover intention of nurses and lead to turnover behaviors. However, very little is known about the role of bidirectional associations between emotional exhaustion and depression/anxiety on turnover intention. This study aimed to examine the associations among depression, anxiety, emotional exhaustion and turnover intention, and to test the role of bidirectional associations between depression, anxiety, and emotional exhaustion on turnover intention among nurses.

**Methods:**

An online multicenter cross-sectional study was conducted in Hunan Province, China, from December 2021 to February 2022. The questionnaire collected data from the Turnover Intention Scale, the Emotional Exhaustion Scale, the Patient Health Questionnaire-2, and the Generalized Anxiety Disorder Scale-2, as well as sociodemographic information. Data analysis was performed by univariate analysis, Pearson correlation analysis, multiple linear regression analysis, and structural equation modeling.

**Results:**

The average turnover intention score among Chinese nurses was 14.34 ± 3.75. The prevalence of depression and anxiety was 25.9% and 22.3%, respectively. Depression (r = 0.378, *P* < 0.001), anxiety (r = 0.391, *P* < 0.001), and emotional exhaustion (r = 0.532, *P* < 0.001) were positively associated with turnover intention. Emotional exhaustion partially mediated the associations between depression/anxiety and turnover intention, with both mediating effects accounting for 60.7%. The mediating ratios of depression/anxiety on the associations between emotional exhaustion and turnover intentions were 17.6% and 16.5%, respectively.

**Conclusions:**

Depression, anxiety, and emotional exhaustion showed significant positive effects on turnover intention among nurses. Emotional exhaustion played a partial mediation role between depression/anxiety and turnover intention, while depression/anxiety played no significant mediation role between emotional exhaustion and turnover intention.

**Supplementary Information:**

The online version contains supplementary material available at 10.1186/s12912-023-01516-1.

## Background

The World Health Organization (WHO) estimates a shortage of 5.7 million nurses worldwide by 2030 [1]. The high turnover rate of nurses will exacerbate the existing issue. The global turnover rates for nurses ranged from 4.5 to 44.3%, with the highest rates occurring in low- and middle-income countries [2]. A national survey in China revealed that the total turnover rate of Chinese nurses was 2.15%, with turnover rates ranging from 3.13 to 4.87% for nurses with less than five years of experience [3]. The healthcare system has been profoundly affected by the high turnover rate of nurses. A systematic review in 2022 indicated that the turnover of nurses could have noneconomic and economic consequences [4]. In terms of noneconomic consequences, it may reduce workshop learning and patient hours per day, increase job demands, contribute to poor mental health, and reduce nursing job satisfaction and patient outcomes (patient satisfaction, patient falls, medical errors, and average length of patient stay). Economically, it can increase medical costs, as turnover costs are three times the average annual salary of nurses. Thus, urgent measures are needed to address the high global turnover rates of nurses in order to improve the quality of nursing care and reduce medical costs.

Turnover intention was considered the most direct predictor of turnover behavior [5]. A rapid systematic review showed that the estimated global turnover intention among nurses was 31.7% [6]. Notably, a cross-sectional study involving 63,947 Chinese nurses revealed a higher turnover intention rate, reaching 63.4% [7]. Understanding the factors and mechanisms that determine nurses’ intentions to leave in order to reduce turnover rates has attracted considerable attention from researchers so far. Pang et al. [8] found that nurses with moderate or severe depressive symptoms had a significantly increased turnover intention, ranging from 2.81 to 4.60 times higher. Additionally, Tabur et al. [9] suggested that higher levels of anxiety among healthcare workers were associated with stronger turnover intentions. While depression and anxiety may have positive effects on turnover intention, it’s important to note that burnout was one of the strongest predictors [10]. A meta-analysis revealed significant associations between burnout and both depression and anxiety with no conclusive overlap; nevertheless, their causal associations are still unclear [11].

However, several studies considered that depression and anxiety were predictors of burnout [12], while others revealed that burnout could also predict depression and anxiety [13, 14]. These findings indicated the possibility of bidirectional associations between depression/anxiety and burnout, suggesting mutual influence and interaction. Burnout has three dimensions: emotional exhaustion, depersonalization, and personal accomplishment. Levante et al. [15] discovered bidirectional associations between the depersonalization dimension of burnout and depression/anxiety. Özkan et al. [16] emphasized that emotional exhaustion had the greatest effect on turnover intention. Furthermore, a systematic review and meta-analysis [17] revealed that the emotional exhaustion dimension of burnout was most strongly associated with depression in nurses. However, little is known about the bidirectional associations between depression/anxiety and emotional exhaustion. Few studies have examined these three factors inside integrated models to predict turnover intention. In a regression model by Tabur et al. [9], depression, anxiety and emotional exhaustion were taken as predictors of turnover intention, but their interconnection was overlooked.

**Fig. 1:**
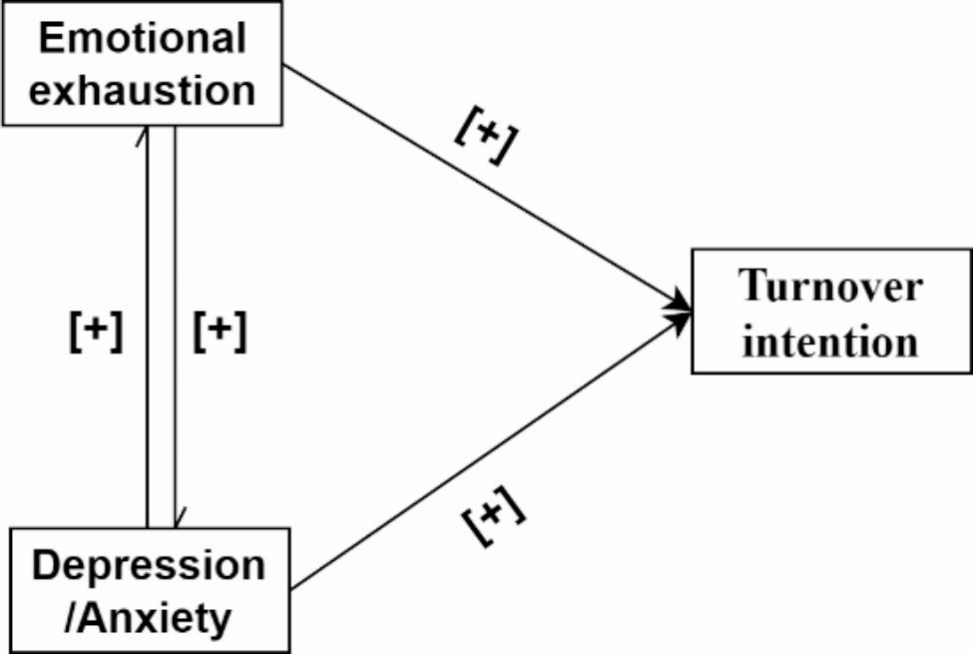
Theoretical framework

The theoretical framework for this study (Fig. 1) focuses on the role of bidirectional associations between depression, anxiety and emotional exhaustion on turnover intention among nurses. According to the Job Demands-Resources (JD-R) model [18], nurses frequently encounter high job demands and insufficient job resources [19, 20], leading to depression, anxiety, and emotional exhaustion, and ultimately a higher intention to leave their jobs. Building upon the Transactional Model of Stress and Coping [21], we hypothesized bidirectional associations among depression, anxiety and emotional exhaustion. Specifically, nurses with elevated levels of depression and anxiety may be more prone to experiencing heightened emotional exhaustion, which is often worsened by negative cognitive patterns and ineffective coping strategies. Conversely, emotional exhaustion, as a result of demanding work environments, may deplete job resources, heightening vulnerability to various stressors and possibly leading to the development of depression and anxiety in nurses. Overall, the aim of this study was to examine the associations among depression, anxiety, emotional exhaustion and turnover intention, and to test the role of bidirectional associations between depression, anxiety, and emotional exhaustion on turnover intention among nurses. The following hypotheses are held:

### Hypothesis 1

Depression, anxiety and emotional exhaustion were the predictors of turnover intention among nurses.

### Hypothesis 2

Emotional exhaustion mediated the association between depression (Model 1), anxiety (Model 2) and turnover intention among nurses: higher levels of depression/anxiety were associated with stronger turnover intention, with higher levels of emotional exhaustion playing a mediating role.

### Hypothesis 3

Depression (Model 3), anxiety (Model 4) mediated the association between emotional exhaustion and turnover intention among nurses: higher levels of emotional exhaustion were associated with stronger turnover intention, with higher levels of depression/anxiety playing a mediating role.

Depression and anxiety were separated into different models for two main reasons. On the one hand, depression and anxiety are distinct mental health issues with potentially different underlying mechanisms and outcomes. For instance, the development of depression may involve complex metabolic pathways, including monoamines and the Hypothalamic–Pituitary–Adrenal (HPA) axis [22]. In contrast, anxiety was primarily associated with neurochemical imbalances such as changes in glutamate and serotonin [23]. Furthermore, these conditions yield different outcomes. Depression is frequently characterized by low mood, reduced motivation, sleep problems, and changes in appetite, which may lead to social isolation and cognitive problems [24]. In contrast, anxiety often appears as heightened nervousness, excessive worry, palpitations, rapid breathing, tense muscles, and social avoidance, all of which can impact concentration and decision-making abilities [25]. By analyzing them separately, we can gain a more comprehensive understanding of their individual effects on turnover intention, thereby shedding light on their unique contributions. On the other hand, it enables us to compare the potential differences in the mechanisms and consequences of depression and anxiety concerning turnover intention. Previous research has shown that coping and cognitive mechanisms related to depression and anxiety can vary among nurses, leading to different turnover intention outcomes [26]. Nurses dealing with depression often lean towards avoidance and social withdrawal as primary coping strategies [27]. They tend to focus more on negative information and engage in rumination [28]. Conversely, nurses dealing with anxiety tend to rely heavily on an excessive attention mechanism [29]. They overly fixate on potential dangers and threats, resulting in catastrophic thought patterns and skewed evaluations [26].

In general, this study can provide a comprehensive understanding of the interconnections between depression, anxiety, emotional exhaustion, and turnover intention in nurses. The bidirectional results may shed light on potential causal associations among these variables. These findings can provide specific evidence on how mental health issues can influence turnover intention, thereby providing nursing administrators with critical information for implementing targeted psychological interventions or services. This can help address psychological issues in the nursing workplace and reduce turnover.

## Methods

### Study design

This study was conducted with nurses from Hunan, China, using a multicenter cross-sectional design, and the results were reported in accordance with the STROBE Statement [30]. The cross-sectional study design allows for a rapid exploration of the potential relationships between depression, anxiety, emotional exhaustion, and turnover intention. The use of structural equation modeling (SEM) offers a preliminary investigation into the role of bidirectional associations between depression, anxiety, and emotional exhaustion on turnover intention. Compared to longitudinal studies, a cross-sectional study is more cost-effective and can determine whether more in-depth research is warranted, thereby guiding and supporting future research endeavors.

### Setting and participants

Participants were recruited from 15 tertiary hospitals located in different geographical areas of Hunan Province, China (Northern Hunan, Western Hunan, Southern Hunan, Central Hunan, and Eastern Hunan) between December 2021 and February 2022. All the hospitals were well-known local comprehensive or general hospitals with over 500 beds. Each hospital has more than 200 nurses capable of providing specialized nursing care at a high level. The inclusion criteria were as follows: (1) registered nurses in China; (2) clinical nurses in tertiary hospitals; and (3) nurses who provide direct care to hospital patients. Exclusion criteria included: (1) nurses with major physical illnesses, such as malignant tumors and acute disease conditions during the study period; (2) inter-hospital intern nurses; (3) intern nurses; and (4) nursing clinical managers (the directors of the nursing department and head nurses).

### Data collection

The online questionnaires were collected using an online platform (https://www.wjx.cn/). After consulting with the nursing managers of the 15 tertiary hospitals included in the study, the nursing managers would receive a link to the online questionnaire, which they would then distribute to the eligible participants of their hospitals via WeChat workgroup at each level, inviting nurses to complete the online questionnaire. To reduce the possibility of selection bias, a comprehensive strategy was used to recruit study participants. Clinical nurses from a variety of departments, including the emergency department, intensive care unit, general wards, operating rooms, and others, were recruited. This recruitment spanned 15 tertiary hospitals across five distinct geographical regions within Hunan Province, China. Additionally, to minimize reporting bias, an anonymous online survey was conducted, and participants were assured of data anonymity, emphasizing that the data would be used solely for scientific research purposes. On the front page of the questionnaire, the objectives, contents, potential benefits, and risks of this survey were described, and participants were then encouraged to provide informed consent online before completing the questionnaire. It took approximately 15 to 20 min to complete the questionnaire, and participation was entirely voluntary. After collecting the questionnaires, the investigators independently analyzed and checked each one online. Multiple approaches were taken to improve online data quality and reduce information bias. After obtaining informed consent, each participant’s IP address was recorded to ensure that they could only complete the questionnaire once. The time limit for completing the survey is 150–3000 s. Too little or too much time were considered invalid data. To prevent missing data, survey responses could only be submitted after all questions had been answered. There were a total of 1205 questionnaires submitted by nurses, 74 of which were deemed of poor quality due to inadequate (59) or excessive (15) completion times. After double-checking and verifying, a total of 1131 valid questionnaires were included in the final analysis, for a valid response rate of 93.9%.

### Sample size

The sample size was determined using multiple statistical approaches. The sample size for multiple linear regression analysis should be greater than 15–25 times the number of independent variables [31]. In this study, there were 10 possible independent variables, and a minimum sample size of 250 was calculated. Sim et al. [32] recommend a minimum sample size of 880 for simple mediation models under the partial mediation condition in SEM with a large indirect effect size, three indicators, and 0.4 loadings in the bootstrap method. Taking into account the invalid questionnaires, raise the sample size by 20%; the minimum sample size for this study was 1100.

### Measures

To reduce measurement bias, all measurement tools used in this study had Chinese versions with good reliability and validity, and clear instructions were provided during the survey.

#### Turnover intention

The Turnover Intention Scale (TI) developed by Michael and Spector [33] was used to measure the intention to leave of employees. The Chinese version of the scale was translated and revised by Dongrong Li and Jingyuan Li [34]. The six-item scale has three dimensions: the possibility of an employee quitting the present job (items 1 and 6), the motivation for employees to find other jobs (items 2 and 3), and the possibility of employees having access to external work (items 4 and 5). A Likert 4-point scale ranging from 1 (never) to 4 (often) was used, with higher scores indicating a stronger intention to leave. The Chinese version had a Cronbach’s α of 0.77 and a content validity rating of 0.76. The scale’s reliability and validity were acceptable among Chinese nurses [35].

#### Emotion exhaustion

A three-item measure scale adapted from the Emotional Exhaustion subscale of the Maslach Burnout Inventory [36] was developed by Boswell et al. [37] to assess emotional exhaustion, which contained three items: “I feel emotionally drained from my work,” “I feel burned out from my work,” and “I feel exhausted when I think about having to face another day on the job.” On a seven-point scale, responses range from strongly disagree (1 point) to strongly agree (7 points). In this study, the Cronbach’s α of the scale was 0.948.

#### Depression and anxiety

The Patient Health Questionnaire-2 (PHQ-2) and Generalized Anxiety Disorder Scale-2 (GAD-2) were used to assess the depression and anxiety of nurses. Each scale consisted of two items and was scored on a 4-point Likert scale, with a total score ranging from 0 to 6. A score of ≥ 3 on each scale indicates signs of depression and anxiety, respectively. The Chinese version of PHQ-2 has acceptable reliability with a Cronbach’s α of 0.727 to 0.785 [38]. The Chinese version of GAD-2 had acceptable reliability with a Cronbach’s α of 0.806 [39].

#### Sociodemographic characteristics

According to previous studies [40, 41], the sociodemographic information included gender, age, education level, self-directed choice of nursing, single status, years of work, professional title, and work unit, which may influence the levels of turnover intention among nurses.

### Data analysis

The statistical software SPSS 26.0 and AMOS 26.0 were used for data analysis. The outcome was the turnover intention score; sociodemographic characteristics were considered potential confounding factors, and depression, anxiety, and emotional exhaustion were used as independent and mediating variables, respectively. Categorical variables were described by frequency and percentage. Values of skewness and kurtosis falling within the range of -2 to + 2 were typically deemed acceptable indicators of a normal univariate distribution [42]. All continuous variables were determined to have a normal distribution due to skewness<|1| and kurtosis<|1| (depression: 0.655, 0.021; anxiety: 0.723, 0.141; emotional exhaustion: 0.137, 0.807; turnover intention: -0.405, -0.509); hence, anxiety, depression, emotional exhaustion and turnover intention were described using the mean ± standard deviation (SD). Then, the independent *t* test and one-way analysis of variance (one-way ANOVA) were used to compare the difference in turnover intention between sociodemographic groups. Welch analysis was used when the variance was not homogeneous. To validate bidirectional associations between depression, anxiety, and emotional exhaustion and further investigate the role of bidirectional associations on turnover intention, the Pearson correlation analysis was utilized to examine the mutual associations between depression/anxiety, and emotional exhaustion, as well as to evaluate their associations with turnover intention. Then, multiple linear regression analysis was performed with turnover intention as the dependent variable to determine whether depression/anxiety, and emotional exhaustion significantly influenced turnover intention. A sensitivity analysis was conducted to compare unadjusted and adjusted models, thereby enhancing the robustness of the study’s findings. Finally, the SEM using the maximum likelihood estimation method was constructed to validate the bidirectional associations between depression/anxiety and emotional exhaustion, and further explore their impacts on turnover intention. In general, the mediating effect was considered to be more than 80% fully mediated, between more than 20% and less than 80% partially mediated, while less than 20% indicates a non-significant mediating effect [43, 44]. *P* < 0.05 was used as the significance level for two-sided tests.

### Ethical considerations

This study was approved by the Institutional Review Board of the Third Xiangya Hospital, Central South University (No. I 22,297).

## Results

### Sociodemographic characteristics

Among the 1131 nurses, the majority were female (1050/92.8%), and the average age was 30.74 ± 6.34 years. Univariate analyses revealed that age, education level, self-directed choice of nursing, single status and work unit were significantly associated with turnover intention among nurses (*P* < 0.05). More details are shown in Table 1.

**Table 1:** Sociodemographic characteristics of the sample (N = 1131)

Variables	N (%)	Turnover intention	*F/t*	*P value*
**Gender**			0.426 ^*t*^	0.670
Male	81(7.2)	14.51 ± 3.47		
Female	1050(92.8)	14.32 ± 3.77		
**Age (years)**			13.180 ^*F*^	< 0.001
<25	189(16.7)	14.44 ± 3.70		
25–44	901(79.7)	14.45 ± 3.73		
>44	41(3.6)	11.41 ± 3.36		
**Education level**			8.900 ^*F*^	< 0.001
Junior college or below	223(19.7)	13.45 ± 4.11		
Bachelor degree	847(74.9)	14.49 ± 3.63		
Master and above	61(5.4)	15.46 ± 3.50		
**Self-directed choice of nursing**			-11.449 ^*t*^	< 0.001
Yes	863(76.3)	13.72 ± 3.73		
No	268(23.7)	16.31 ± 3.06		
**Single status**			3.643 ^*t*^	< 0.001
Yes	305(27.0)	15.00 ± 3.65		
No	826(73.0)	14.09 ± 3.76		
**Years of work (years)**			2.568 ^*F*^	0.077
<5	313(27.7)	14.49 ± 3.73		
5–10	347(30.7)	14.60 ± 3.64		
>10	471(41.6)	14.04 ± 3.83		
**Professional title**			1.763 ^*F*^	0.152
Nurse	184(16.3)	14.13 ± 3.90		
Nurse practitioner	455(40.2)	14.59 ± 3.69		
Nurse-in-charge	476(42.1)	14.22 ± 3.74		
Associate chief nurse or above	16(1.4)	12.94 ± 3.57		
**Work unit**			10.790 ^*F*^	< 0.001
Emergency department	286(25.3)	14.47 ± 3.80		
Intensive care unit	91(8.0)	15.68 ± 3.16		
General wards	453(40.1)	14.54 ± 3.78		
Operating room	138(12.2)	14.26 ± 3.33		
Others	163(14.4)	12.84 ± 3.80		

* F*, one-way analysis of variance, *t*, independent *t* test

### Depression, anxiety, emotional exhaustion and turnover intention of nurses

The prevalence of depression and anxiety among nurses was 25.9% and 22.3%, respectively. The average scores for depression, anxiety, emotional exhaustion and turnover intention were 1.92 ± 1.59, 1.90 ± 1.60, 11.55 ± 4.84 and 14.34 ± 3.75, respectively. The mean scores of each turnover intention dimension, from highest to lowest, were as follows: 5.50 ± 1.27 (the possibility of employees having access to external work), 4.45 ± 1.66 (the motivation for employees to find other jobs) and 4.38 ± 1.61 (the possibility of an employee quitting the present job).

### Associations among depression, anxiety, emotional exhaustion and turnover intention

Table 2 showed the Pearson correlation analysis results. Emotional exhaustion showed a moderately positive correlation with both depression (*r* = 0.521) and anxiety (*r* = 0.538). Turnover intention showed a mildly significant positive correlation with both depression (*r* = 0.378) and anxiety (*r* = 0.391), as well as a moderately significant positive correlation with emotional exhaustion (*r* = 0.532).

**Table 2:** Correlations between depression, anxiety, emotional exhaustion and turnover intention (N = 1131)

	Depression	Anxiety	Emotional exhaustion
Depression			
Anxiety	0.847^***^		
Emotional exhaustion	0.521^***^	0.538^***^	
Turnover intention	0.378^***^	0.391^***^	0.532^***^

^***^P < 0.001

Both unadjusted and adjusted models of multiple linear regression analysis showed significant associations between depression/anxiety, emotional exhaustion and turnover intention. Depression, anxiety, and emotional exhaustion were positive predictors of turnover intention among nurses. Results are detailed in Table 3.

**Table 3:** Multiple linear regression analysis of turnover intention among nurses (N = 1131)

Independent variable	*β (95% CI)*			
	Model 1a	Model 1b	Model 2a	Model 2b
Emotional exhaustion	0.459 (0.311, 0.400) ^***^	0.402 (0.266, 0.356) ^***^	0.452 (0.305, 0.359) ^***^	0.395 (0.260, 0.352) ^***^
Depression/Anxiety	0.138 (0.192, 0.463) ^***^	0.139 (0.196, 0.462) ^***^	0.148 (0.210, 0.483) ^***^	0.147 (0.212, 0.479) ^***^
Adjusted R^2^	0.295	0.329	0.297	0.330

Models 1a and 1b: depression and emotional exhaustion were used as independent variables

Models 2a and 2b: anxiety and emotional exhaustion were used as independent variables

Models 1a and 2a, unadjusted models; Models 2b and 2b, adjusted for age, education level, self-directed choice of nursing, single status and work unit

*β*, standardized regression coefficient; *95%CI*, 95% confidence interval for regression coefficient

^***^P < 0.001

### The role of bidirectional associations between depression, anxiety, and emotional exhaustion on turnover intention

**Fig. 2:**
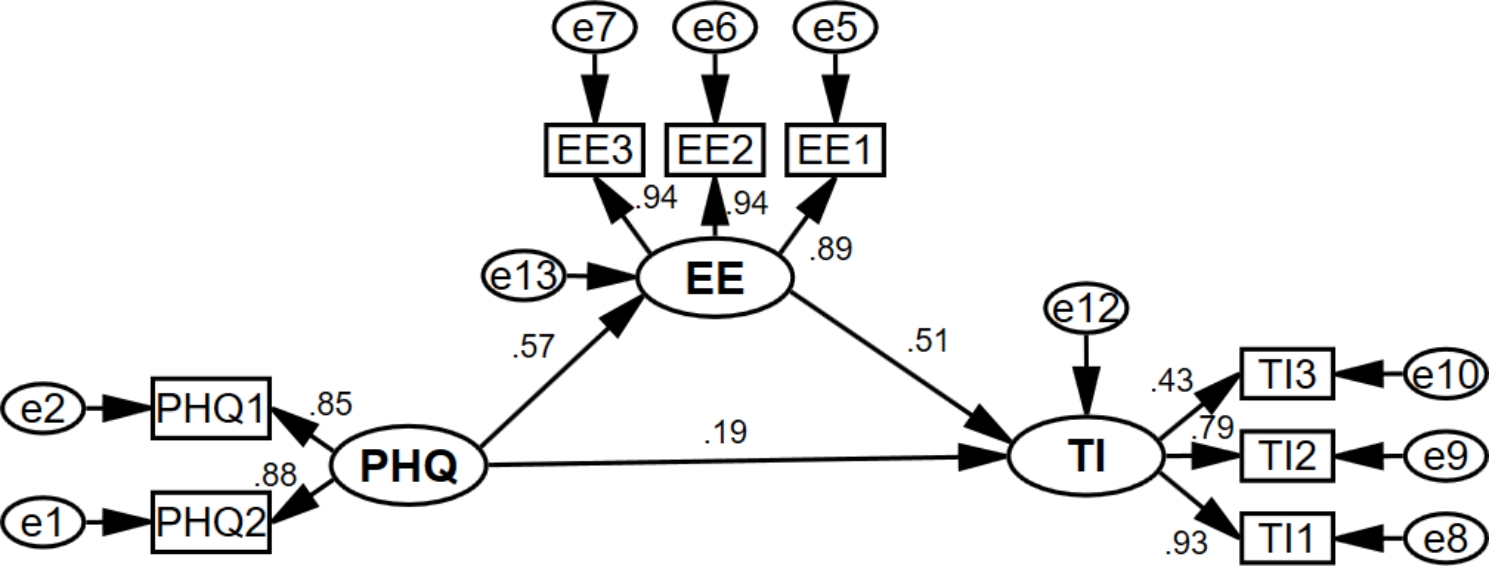
Model 1

PHQ: Depression; PHQ1: “Little interest or pleasure in doing things”; PHQ2: “Feeling down, depressed, or hopeless”; EE: Emotional Exhaustion; EE1: “I feel emotionally drained from my work”; EE2: “I feel burned out from my work”; EE3: “I feel exhausted when I think about having to face another day on the job”; TI: Turnover intention; TI1: the possibility of an employee quitting the present job; TI2: the motivation for employees to find other jobs; TI3: the possibility of employees having access to external work.

**Fig. 3:**
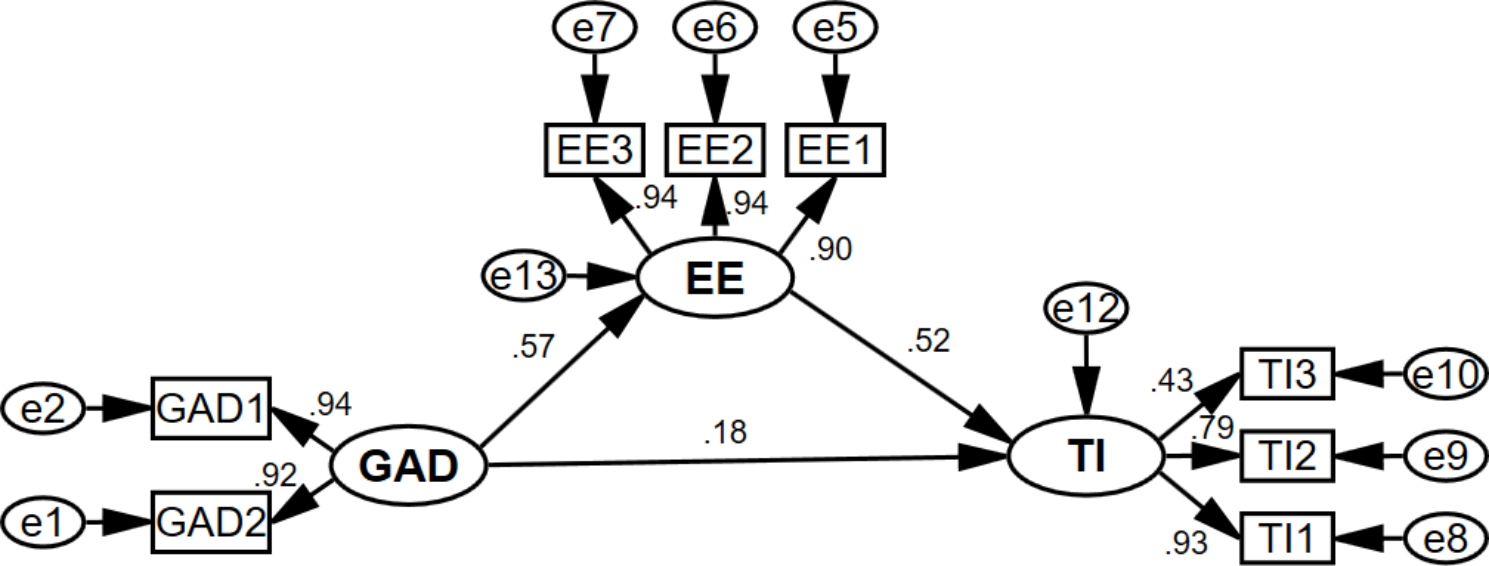
Model 2

GAD: Anxiety; GAD1: “Feeling nervous, anxious or on edge”; GAD2: “Not being able to stop or control worrying”; EE: Emotional Exhaustion; EE1: “I feel emotionally drained from my work”; EE2: “I feel burned out from my work”; EE3: “I feel exhausted when I think about having to face another day on the job”; TI: Turnover intention; TI1: the possibility of an employee quitting the present job; TI2: the motivation for employees to find other jobs; TI3: the possibility of employees having access to external work.

**Fig. 4:**
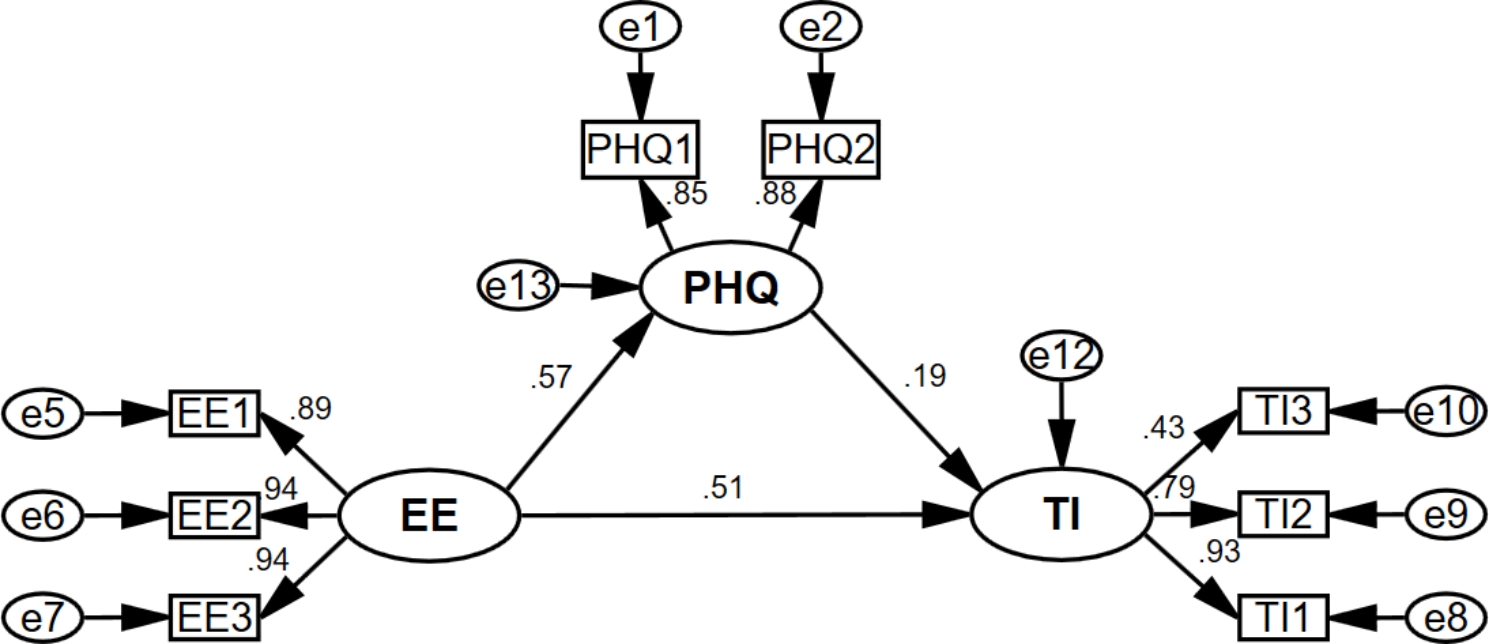
Model 3

EE: Emotional Exhaustion; EE1: “I feel emotionally drained from my work”; EE2: “I feel burned out from my work”; EE3: “I feel exhausted when I think about having to face another day on the job”; PHQ: Depression; PHQ1: “Little interest or pleasure in doing things”; PHQ2: “Feeling down, depressed, or hopeless”; TI: Turnover intention; TI1: the possibility of an employee quitting the present job; TI2: the motivation for employees to find other jobs; TI3: the possibility of employees having access to external work.

**Fig. 5:**
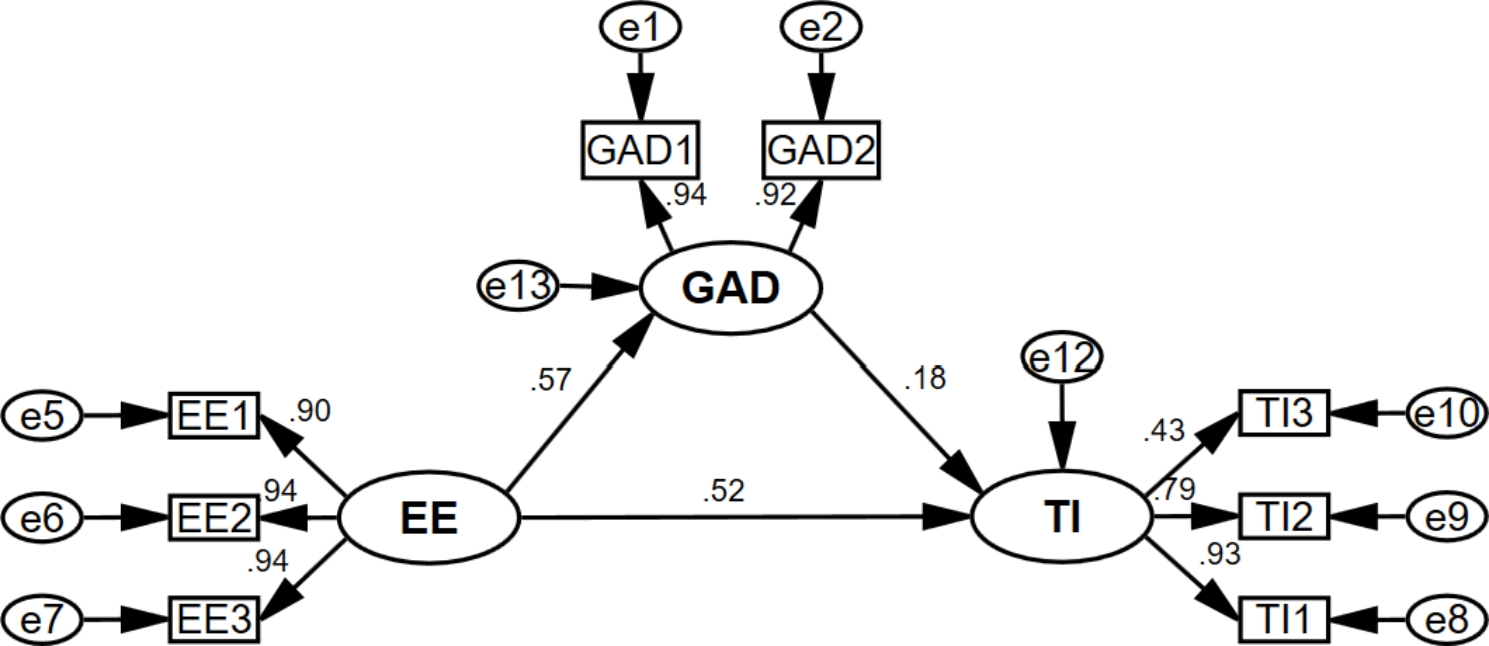
Model 4

EE: Emotional Exhaustion; EE1: “I feel emotionally drained from my work”; EE2: “I feel burned out from my work”; EE3: “I feel exhausted when I think about having to face another day on the job”; GAD: Anxiety; GAD1: “Feeling nervous, anxious or on edge”; GAD2: “Not being able to stop or control worrying”; TI: Turnover intention; TI1: the possibility of an employee quitting the present job; TI2: the motivation for employees to find other jobs; TI3: the possibility of employees having access to external work.

To test the hypotheses, models 1–4 were constructed, as shown in Figs. 2, 3, 4 and 5. Each model’s model fit indices demonstrated good fit (**Appendix 1)**. Depression (β = 0.483, for the total effect) and anxiety (β = 0.483, for the total effect) were significantly and positively associated with turnover intention. Model 1 and Model 2 revealed that emotional exhaustion partially mediated the association between depression/anxiety and turnover intention, both with a mediation effect ratio of 60.7%. Meanwhile, emotional exhaustion (β = 0.620, for the total effect) was significantly and positively associated with turnover intention. Model 3 showed that the mediating ratio of depression was 17.6%, and Model 4 showed that the mediating ratio of anxiety was 16.5%. See more details in Table 4.

**Table 4:** The role of bidirectional associations between depression, anxiety, and emotional exhaustion on turnover intention (N = 1131)

Estimate	B	β	*95%CI*
*Total effect*			
Model 1: Depression→TI	0.365	0.483	(0.423, 0.540) ^***^
Model 2: Anxiety→TI	0.350	0.483	(0.423, 0.540) ^***^
Model 3: EE→TI ^a^	0.209	0.620	(0.571, 0.664) ^***^
Model 4: EE→TI^b^	0.209	0.620	(0.571, 0.664) ^***^
*Indirect effect*			
Model 1: Depression→EE→TI	0.222	0.293	(0.245, 0.346) ^***^
Model 2: Anxiety→EE→TI	0.212	0.293	(0.245, 0.346) ^***^
Model 3: EE→Depression→TI	0.037	0.109	(0.064, 0.155) ^***^
Model 4: EE→Anxiety→TI	0.050	0.102	(0.061, 0.141) ^***^
*Direct effect*			
Model 1: Depression→TI	0.144	0.190	(0.109, 0.268) ^***^
Model 2: Anxiety→TI	0.137	0.190	(0.109, 0.268) ^***^
Model 3: EE→TI ^a^	0.212	0.577	(0.441, 0.577) ^***^
Model 4: EE→TI ^b^	0.216	0.518	(0.450, 0.582) ^***^

^a^ depression as a mediating variable; ^b^ anxiety as a mediating variable; EE: emotional exhaustion; TI: turnover intention; B, path coefficient; β, standardized path coefficient; 95%*CI*, 95% confidence interval for the standardized path coefficient

^***^P < 0.001

## Discussion

To our knowledge, this is the first study to test the role of bidirectional associations between depression, anxiety, and emotional exhaustion on turnover intention among nurses using a multicenter cross-sectional design. The results demonstrated that turnover intention was significantly and positively associated with depression, anxiety, and emotional exhaustion. Emotional exhaustion partially mediated the association between depression/anxiety and turnover intention, and depression/anxiety played a non-significant mediating role between emotional exhaustion and turnover intention.

The results showed that turnover intention has significant and positive associations with depression, anxiety, and emotional exhaustion, supporting hypothesis 1. This was consistent with some studies while contradicting others. Several studies have confirmed the positive associations between depression, anxiety, emotional exhaustion and turnover intention [8, 45, 46]. However, Tabur et al. [9] revealed that depression and general anxiety were not predictors of turnover intention among healthcare professionals. These results appear inconsistent with those of this study, which can largely be attributed to the discrepancies in the statistical analysis method. Due to the significant correlation between depression and anxiety scores, statistically significant results could change when depression and anxiety were entered into the same model simultaneously [47]. This study also emphasized that emotional exhaustion had a larger association with turnover intention than depression/anxiety. This is mostly due to the fact that emotional exhaustion stems from job-related negative experiences rather than life domains [48], which may directly lead to an intention to quit [16]. Meanwhile, this study revealed moderate associations between depression, anxiety, and emotional exhaustion, which was consistent with previous systematic reviews [17, 49].

The results indicated that emotional exhaustion played a partial mediating role between depression/anxiety and turnover intention, both with a mediation effect ratio of 60.7%, supporting hypothesis 2. This was partially consistent with previous research results indicating that depression/anxiety could increase emotional exhaustion [50, 51], which directly leads to increased turnover intention [52]. Analyzing potential mechanisms, it is possible that individuals experiencing emotional exhaustion typically have a decreased ability to regulate negative emotions [53], feel physically and mentally exhausted, and experience feelings of loneliness, which makes it difficult for them to receive support from others and effectively cope with job demands [54]. As a result, they are more susceptible to the impact of work-related stress and negative events, thus moderating the effects of depression and anxiety on turnover intention and increasing the likelihood of turnover. Similarly, it was discovered that emotional exhaustion may have medicating effects between various mental health problems and turnover intention. Shah et al. discovered that emotional exhaustion mediates the relationship between COVID-19-related job stress and turnover intention [46]. Santo et al. [55] demonstrated that emotional exhaustion mediated the effect of emotional dysregulation on turnover intention. All of these similar findings underline the key role of emotional exhaustion in turnover intention. Furthermore, this study discovered the significant effects of depression, anxiety, and emotional exhaustion on the turnover intention of nurses, highlighting the great value of early detection and management in reducing turnover intention.

Surprisingly, the mediation effect ratios of depression and anxiety between emotional exhaustion and turnover intention were both less than 20%, indicating non-significant mediating effects and suggesting that hypothesis 3 does not hold [43, 44]. A possible explanation for the lack of significance was that emotional exhaustion could be an early indicator of turnover intention, while depression and anxiety may develop in the later stages following emotional exhaustion. Although some cross-sectional research showed that emotional exhaustion could increase the risk of depression [56, 57], Chen & Meier et al. [17] discovered that the correlation between emotional exhaustion and depression increased as nurses gained more experience and age, and several longitudinal studies further revealed that emotional exhaustion could predict depressive symptoms over periods of 3- and 4-years [13, 14]. All these findings indicated that emotional exhaustion may be an early symptom that precedes depression and anxiety. Emotional exhaustion reflects negative experiences and stress individuals face in the work environment, and it may serve as a precursor to depression and anxiety. Therefore, due to the high overlap between emotional exhaustion and depression/anxiety [58–60], when considering emotional exhaustion, the independent contributions of depression/anxiety in explaining turnover intention may be relatively small. Therefore, emotional exhaustion may play a partial mediating role between depression/anxiety and turnover intention, but emotional exhaustion may not directly influence turnover intention through depression and anxiety. This suggests that emotional exhaustion may play a significant mediating role in the development of turnover intention, while depression and anxiety may further exacerbate turnover intention in the later stages following emotional exhaustion. Therefore, longitudinal studies are required to examine how the emotional exhaustion-depression/anxiety associations affect the turnover intention of nurses over time.

### Implication

This study highlights the mediating role of emotional exhaustion in the relationship between depression/anxiety and turnover intention among nurses, which emphasized the critical importance of addressing nurses’ mental health, particularly in managing emotional exhaustion issues within the nursing work environment, to alleviate turnover intention. The findings can provide guidance for policy-making within hospital human resources departments, offering several targeted practical recommendations. On the one hand, a supportive nursing work environment can be established through various approaches to alleviate job-related stress and enhance mental well-being. These methods include implementing flexible scheduling [61], establishing open communication channels [62], providing resiliency training, cultivating teamwork and spiritual support [63], and conducting stress management workshops [64]. On the other hand, a variety of interventions can enhance effective emotional coping strategies and emotion regulation skills for nurses. These interventions encompass mental health support and counseling, self-care workshops, activities like yoga and massage, practices such as mindfulness and meditation, as well as training in stress management and communication skills [64]. These strategies can not only alleviate human resource burdens and empower hospital administrators to implement focused mental health management and preventive measures, but also aid nurses in experiencing diminished negative emotions and emotional exhaustion. As a result, this could ultimately lead to a reduction in their intention to leave their positions.

### Limitations

There are several limitations. First, nurses from Hunan Province, China, were selected, which may have resulted in selection bias. It may affect the generalizability of this study’s findings. To capture a more representative sample, the recruitment strategy was designed to encompass clinical nurses from a diverse array of departments, including the emergency department, intensive care unit, general wards, operating rooms, and other areas. To increase diversity and inclusiveness, the recruitment approach extended across 15 tertiary hospitals located within five distinct geographical regions across Hunan Province, China. Second, despite the bidirectional hypotheses set forth in this study, causality could not be directly proven, and future longitudinal studies are required to clarify the causal associations between these variables in this model. Finally, other dimensions of burnout, such as depersonalization and personal accomplishment, were not included. Further pathway analyses could be performed to explore the role of each burnout dimension.

## Conclusion

Depression, anxiety, and emotional exhaustion showed significant positive effects on turnover intention among nurses. Emotional exhaustion played a partial mediation role between depression/anxiety and turnover intention, while depression/anxiety played no significant mediation role between emotional exhaustion and turnover intention. The findings may help nursing leaders and managers better understand the mechanisms by which depression, anxiety and emotional exhaustion contribute to turnover intention and the key role of reducing emotional exhaustion in alleviating turnover intention among nurses. Nursing managers should take effective measures to identify and address psychological problems to improve turnover among nurses, with a particular emphasis on enhancing emotion regulation skills and reducing emotional exhaustion.

### Electronic supplementary material

Below is the link to the electronic supplementary material.

Supplementary Material 1

## Data Availability

The data can be obtained by contacting the correspondence author.
