# Supplementary material for: The role of bidirectional associations between depression, anxiety, and emotional exhaustion on turnover intention among nurses: a multicenter cross-sectional study in China
Source: BMC Nurs. 2023 Oct 3;22:350. doi: 10.1186/s12912-023-01516-1 (PMC10548568; doi:10.1186/s12912-023-01516-1)
Supplement: Supplementary file 1 — Supplementary Material 1 [file 12912_2023_1516_MOESM1_ESM.docx]

**Appendix 1** **|** Fit indices of structural equation models (N = 1131**)**

| **Parameters** | **χ^2^/df** | **RMSEA** | **GFI** | **AGFI** | **CFI** | **IFI** | **TLI** | **PCFI** | **PNFI** |
| --- | --- | --- | --- | --- | --- | --- | --- | --- | --- |
| Model 1 | 3.838 | 0.050 | 0.986 | 0.970 | 0.992 | 0.992 | 0.987 | 0.602 | 0.601 |
| Model 2 | 3.652 | 0.048 | 0.987 | 0.972 | 0.993 | 0.993 | 0.989 | 0.603 | 0.602 |
| Model 3 | 3.838 | 0.050 | 0.986 | 0.970 | 0.992 | 0.992 | 0.987 | 0.602 | 0.601 |
| Model 4 | 3.652 | 0.048 | 0.987 | 0.972 | 0.993 | 0.993 | 0.989 | 0.603 | 0.602 |
| Standard values | <5 | <0.08 | >0.90 | >0.90 | >0.90 | >0.90 | >0.90 | >0.50 | >0.50 |

χ^2^/df, Chi-square value degrees of freedom ratio; RMSEA, Root Mean Square Error of Approximation; GFI, Goodness of Fit Index; AGFI, Adjusted Goodness of Fit Index; CFI, Comparative Fit Index; IFI, Incremental Fit Index; TLI, Tucker-Lewis Index; PCFI, Parsimonious Comparative Fit Index; PNFI, Parsimonious Normed Fit Index.
